# The future of Genesis science

**DOI:** 10.1111/maps.13266

**Published:** 2019-02-25

**Authors:** D. S. Burnett, A. J. G. Jurewicz, D. S. Woolum

**Affiliations:** ^1^ Division of Geological and Planetary Sciences California Institute of Technology Pasadena California 91125 USA; ^2^ Center for Meteorite Studies/School of Earth and Space Exploration Arizona State University Tempe Arizona 85287–1404 USA; ^3^ Department of Earth Sciences Dartmouth College Hanover New Hampshire 03755 USA; ^4^ Department of Physics California State University Fullerton California 92831 USA

## Abstract

Solar abundances are important to planetary science since the prevalent model assumes that the composition of the solar photosphere is that of the solar nebula from which planetary materials formed. Thus, solar abundances are a baseline for planetary science. Previously, solar abundances have only been available through spectroscopy or by proxy (CI). The Genesis spacecraft collected and returned samples of the solar wind for laboratory analyses. Elemental and isotopic abundances in solar wind from Genesis samples have been successfully measured despite the crash of the re‐entry capsule. Here we present science rationales for a set of 12 important (and feasible postcrash) Science and Measurement Objectives as goals for the future (Table 1). We also review progress in Genesis sample analyses since the last major review (Burnett 2013). Considerable progress has been made toward understanding elemental fractionation during the extraction of the solar wind from the photosphere, a necessary step in determining true solar abundances from solar wind composition. The suitability of Genesis collectors for specific analyses is also assessed. Thus far, the prevalent model remains viable despite large isotopic variations in a number of volatile elements, but its validity and limitations can be further checked by several Objectives.

## Introduction

### Purpose of the Genesis Mission

The goal of Genesis sample analysis is to determine the average isotopic and elemental compositions of the solar system at levels of precision required for 21st century planetary science by measuring the composition of the solar wind. The quantitative goals for Genesis are to improve solar elemental abundances by a factor of three from photosphere spectroscopic values. For isotopes, prior to Genesis, solar isotopic abundances were virtually unknown, except for He, Ne, and Ar. Genesis isotope precision goals vary with the element, but 10 permil (2 sigma) is acceptable, although better is highly desirable.

### Genesis, Photosphere, and CI Chondrite Abundances

The elemental composition of the solar nebula is commonly assumed to have been uniform and to have been retained by the solar photosphere, the outer portion of the Sun from which visible light arises. Compositional changes due to thermonuclear reactions in the core of the Sun have not affected the photosphere. Thus, the photospheric composition is required for use with cosmochemical models (Burnett 2013). However, spectroscopic measurements of the photosphere (e.g., Asplund et al. 2009) do not have the accuracy needed for many cosmochemical applications. As a proxy for solar composition, planetary scientists currently use analyses of CI chondrites (e.g., Palme et al. 2014). CI and spectroscopic photospheric abundances usually agree. CI abundances are a *valid* representation of solar composition *only* to the extent that they agree with the spectroscopic abundances. The uncertainties in adopted abundances are not the small analytical accuracy of the CI analyses but the relatively large uncertainties in the spectroscopic measurements. CI chondrites are, moreover, very complicated rocks (e.g., McSween 1993) with many opportunities for elemental fractionation during their formation processes.

In terms of elemental abundances, why is it important that the composition we use as a baseline is truly solar and not CI? Much of the cosmochemical literature is based on CI‐normalized abundances. These are convenient representations of compositional data that indicate whether the composition of the sample deviates from the initial solar nebula composition, assuming that CI abundances are identified with solar composition; otherwise the CIs are just an arbitrary reference. Differences in CI‐normalized abundances among C chondrite groups (as well as among H chondrites, which have similar major element composition) can be small, around 10% or less for some elements (Palme et al. 2015), but also large, factors of two to three for moderately volatile elements (Palme et al. 2014). In the case of factors of two to three differences, 10–20% errors in solar spectroscopic abundances may not significantly impede interpretations, but the spectroscopic photospheric abundances of many volatile trace elements have much larger errors. Genesis solar wind abundances are more valid than CI abundances and more accurate than spectroscopic photospheric abundances.

### Solar Wind Collection

Genesis captured and returned samples of solar matter in the form of the solar wind. Most collection was passive: a variety of collector materials (Jurewicz et al. 2003) were exposed to the solar wind, and the momentum of the impacting solar wind ions implanted them sufficiently deep for retention. In addition to a fixed array that collected a bulk solar wind sample, deployable arrays were included to obtain separate samples of distinguishable types (“regimes”) of solar wind (H: high speed solar wind from coronal holes; E: coronal mass ejections; and L: low speed wind from the release of plasma on closed magnetic field loops; Neugebauer et al. 2003). The appropriate array was deployed based on signals from “SW monitors” (Barraclough et al. 2003). A major use of the regime samples is to help understand the fractionation (both elemental and isotopic) caused by the processes of extraction and acceleration of ions from the Sun during solar wind formation. Corrections for this fractionation are needed to derive solar abundances from solar wind data. An electrostatic lens (“Concentrator”) focused solar wind for masses less than about 40, achieving an average concentration factor of about 20 on four “targets,” each made of one of three materials (Wiens et al. 2013; Burnett 2013).

Details on the collector materials and their current state are given in Jurewicz et al. (2003) and https://curator.jsc.nasa.gov/genesis/index.cfm. The Genesis Curator (Johnson Space Center) also curates samples of spacecraft materials and the contaminating dirt from the Utah Test and Training Range.

### Science Versus the Crash

Because the parachute failed to open on re‐entry, the Genesis Sample Return Capsule breached on impact, resulting in major shattering and contamination of collector materials. Fortunately, the solar wind is embedded below the surface of the collectors and retained at depth. Several collectors remained intact: specifically, three of the four Concentrator targets, the bulk metallic glass collector, one bulk‐array wafer, and the two “kidney” collectors (Jurewicz et al. 2003; Wiens et al. 2013).

Much of the early, high‐impact science was gleaned from the Concentrator targets. Measurements of the isotopic compositions of O (McKeegan et al. 2011) and N (Marty et al. 2011) showed that the isotopic compositions of these elements in the Sun was different from that in materials from the terrestrial planets. Analyses of noble gases were relatively unaffected by terrestrial contamination, and several important projects were executed essentially as planned (e.g., Heber et al. 2009a; Meshik et al. 2007; Grimberg et al. 2006; Wieler 2016). He, Ne, and Ar regime measurements showed conclusively, for the first time, that isotopes in the solar wind were fractionated (Heber et al. 2012a).

In contrast, the more delicate array collector array wafers were devastated (Stansbery 2005). With one exception, the impact broke array collectors, in many cases into fragments too small to use effectively. Of the (10000+) array collector fragments recovered with areas greater than 10 mm^2^, many were scratched, pitted, and contaminated by dirt and spacecraft debris (Allton et al. 2005). Array collectors were to be used to measure heavy, low concentration, elements (Ir, Nd, Sm, etc.) plus C isotopes by analyzing large (10–50 cm^2^) areas. Damage to these important collectors has slowed the achievement of Genesis science goals. However, as new sample handling, cleaning, and analytical techniques are perfected, important Genesis science is continuing to be gleaned, even in this nonideal situation (cf. Burnett et al. 2017).

## Future Opportunities in Genesis Science

### Revised Science and Measurement Objectives

To implement the original Genesis Science Objectives, we established a prioritized list of 19 specific “Measurement Objectives,” important and feasible measurements (Burnett 2013). Both crash‐derived issues and advances in techniques and instrumentation call for revision of this list. Our revised set of specific Science and Measurement Objectives is given in Table 1.

**Table 1:** Science and measurement objectives (bulk solar wind unless otherwise specified)

	Specific science objectives	Measurement objectives	Feasibility
1	Improve measurements of SW isotopic fractionation to test possibility that solar O isotopic composition is not on CAI line.	Mg isotopic composition.	Feasible; measurements in progress by several teams.
2	Measure the average solar nebula composition for elements having a low first ionization potential (FIP). These are the rock‐forming elements that make up the terrestrial planets.	Abundances of elements with low FIP, especially in high speed solar wind, which has lowest fractionation.	Feasible for elements lighter than Ni (many require only better analytical standards). Heavier elements require improved techniques.
3	Test for systematic differences in isotopic compositions between Sun and planetary materials.	Isotopic compositions of nonvolatile elements heavier than Ar, specifically Fe.	Fe should be feasible; development required for other elements.
4	Understand the origin and evolution of lunar volatiles.	C isotopic composition.	Development needed, but probably feasible.
5	Test the validity of using the composition of CI chondritic meteorites as a proxy for average solar composition.	Compare SW and CI abundances for as many elements as possible; special emphasis on Mn, Rb, and Ga.	All analyses for task 2 count for this Objective as well. Mn, Rb feasible. Ga needs additional development.
6	Significantly improve knowledge of the average composition of the solar nebula for elements of *high* first ionization potential.	Measure C, N, O abundances in Genesis regime samples, especially high speed.	Feasible; work in progress.
7	Test if either nebular gases or dust were preferentially accreted to the Sun by planetary processes.	Abundances of Se, Br, Kr, Rb, Sr.	Kr already measured. Br, Rb feasible. Se, Sr need additional development.
8	Test for ion‐neutral induced chemical fractionations in the formation of solar system.	Abundances of K, Na, Rb.	Feasible. K, Na in progress.
9	Improved constraints on volatile depletion in formation of chondritic meteorites and the terrestrial planets.	Abundances of B, F, Cl, S, Zn, Se, Br.	F, Cl, S, Br probably feasible; B, Zn, Se need additional development. Major Zn surface contamination problems.
10	Constrain the flux of late accreting planetesimals to the Sun and the amount of thermonuclear processing of the solar photosphere.	Li, Be, B abundances and isotopic compositions.	Development needed but should be feasible.
11	Investigate how planetary materials were modified by the intense solar flare and solar wind exposure caused by the early Sun.	Solar wind radioactive nuclei; F fluence.	Lid foils for radioactive elements were severely contaminated in crash, but considerable progress has been made in cleaning. F probably feasible.
12	Evaluate effects of solar gravitational settling on solar abundances.	^44^C/^40^Ca ratio. Compare relative abundances of heavy (e.g., Ir) and light element (Co, Ni) ratios of siderophile elements with those for CI chondrites.	Feasible overall; Ca isotope ratio difficult. Development of techniques for heavy siderophile elements other than Ir highly desirable.

### Elaboration of Science Objectives

The following paragraphs give a more detailed science discussion of the Objectives from Table 1. Although a prioritized list, implementation should be feasibility driven; e.g., work on Objective 7 should not wait until Objective 1 is done, etc. Gravitational settling from the solar convective zone potentially affects several Objectives, and in this specific case, all discussion is deferred until Objective 12.

#### Objective 1: Mg Isotopes

**Figure 1:**
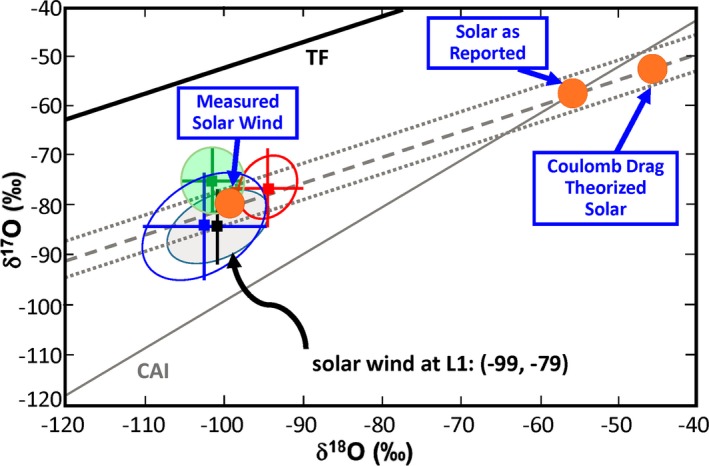
Figure modified from McKeegan et al. (2011). The measured Solar Wind O isotopic composition is precise, but differs from the solar ratios because of isotopic mass fractionation during solar wind acceleration which results in a decrease in ^18^O/^16^O. The direction of the mass dependent fractionation correction is shown by the dashed lines. The Inefficient Coulomb Drag model (Bodmer and Bochsler 2000) prediction lies to the high ^18^O/^16^O side of the CAI line, on which a wide variety of meteoritic materials lie. The adopted Solar as Reported composition on the CAI line was regarded as the most plausible value and is within the uncertainties of the theoretical prediction. [Color figure can be viewed at wileyonlinelibrary.com]

The “CAI line” is a linear trend in the oxygen three‐isotope plot (Fig. 1) based on analyses of a large number of meteoritic samples (mostly Ca‐Al‐rich inclusions; CAIs) which shows an approximately constant ^17^O/^18^O ratio with varying amounts of ^16^O. The solar wind O isotopic composition is precisely measured (McKeegan et al. 2011). *As measured*, the solar wind O isotopic composition does not lie on the CAI line (Fig. 1). The predicted Sun‐solar wind fractionation from Coulomb Drag theory is 28‰/amu (Bodmer and Bochsler 2000) which lies to the high ^18^O/^16^O side of the CAI line (Fig. 1). Use of Coulomb Drag theory is justified because it closely predicts *differences* in the isotopic compositions of He, Ne, and Ar between the high‐ and low‐speed regime samples (Heber et al. 2012a). McKeegan et al. (2011) proposed a fractionation correction such that solar O lies on the CAI line in the O three‐isotope plot (Fig. 1). The adopted fractionation to correct the measured solar wind to the CAI line is 21.5 ± 1.5‰/amu.

Why would it matter if the solar O isotopic composition is significantly different from the CAI line (Fig. 1)? Even uncorrected, the solar wind O isotopic composition (McKeegan et al. 2011) shows that the solar O isotopic composition is very ^16^O‐rich relative to most inner solar system materials. This major difference is explained by self‐shielding models (e.g., Lee et al. 2008; Lyons 2012; Nuth et al. 2014), strongly supported by the observation of very ^16^O‐poor materials in the hydrated matrices of Semarkona and Acfer 094 (Sakamoto et al. 2007). But, if the CAI line (Fig. 1) is a mixing line between ^16^O‐poor terrestrial planet materials and an ^16^O‐rich component, the ^16^O‐rich component would *not* be solar if the Genesis solar O isotopic composition did not lie on the CAI line, showing that the solar nebula was not isotopically homogeneous (at least having components of varying ^17^O/^18^O ratios) and that the pre‐self‐shielding O isotope reservoir for materials on the CAI line was not solar.

We can provide additional constraints on whether the solar O isotopic composition lies on the CAI line by measuring solar wind Mg isotopes. This measurement differs from all other Genesis isotopic studies. Here we are not searching for systematic isotopic differences between the Sun and terrestrial planet materials. Rather, it is *assumed* that the Sun and the Earth have the same isotopic composition and that the differences between solar wind and terrestrial Mg are due to isotopic fractionation in the formation of the solar wind. Unlike O, for which there are large variations among planetary materials, the isotopic composition of Mg can be reliably assumed to be the same for the Earth as for the Sun. (This assumption can be checked; see Objective 3.) A literature search revealed 17 papers in the period 2010–2016 on Mg isotopic compositions in terrestrial igneous or high‐grade metamorphic rocks. Relative to either MORB or chondrites, the vast majority of samples deviated in ^26^Mg/^24^Mg by <0.5‰: Measured in the same study, ^26^Mg/^24^Mg in MORB and bulk chondrite samples differ by at most 0.1‰ (e.g., Bourdon et al. 2010; Schiller et al. 2010). Relative to MORB, analyses of a large number of mantle samples (e.g., fig. 6 of Xiao et al. 2013) have ^26^Mg/^24^Mg differences well within ±0.5‰. Similarly, continental crustal rocks are higher in ^26^Mg/^24^Mg from MORB by at most 0.5‰ (e.g., fig. 6 of Telus et al. 2012). The above variations are real, representing physical/chemical isotope fractionation, presumably mass dependent. Nevertheless, the variations are small compared to the predicted 10‰/amu from Coulomb Drag theory (Bochsler 2000), and within the ranges quoted, these variations represent the average Mg isotopic composition of the Earth. Meteoritic Mg isotope data are best discussed in terms of ^25^Mg/^24^Mg because of contributions of radiogenic ^26^Mg from ^26^Al decay. Relatively large deviations in ^25^Mg/^24^Mg have been found at small scales. For example, Ca‐Al‐rich inclusion (CAI) materials exhibit large variations (up to 30‰) in ^25^Mg/^24^Mg due to evaporation effects (e.g., Davis and Richter 2014). Smaller but similar evaporation effects may show up in individual chondrule ^25^Mg/^24^Mg analyses (Villeneuve et al. 2011; Bouvier et al. 2013) which show variations as large as 1.5‰.

Summarizing, excepting CAIs, Mg isotopic variations among major inner solar system reservoirs appear to be <1‰/amu. Thus, any isotopic difference between the Earth and the solar wind greater than about 1‰ in ^26^Mg/^24^Mg can be equated to differences between the Sun and the solar wind, i.e., the amount of solar wind isotopic fractionation of Mg, which can then be used to estimate the O fractionation.

The Coulomb Drag model (e.g., Bochsler 2000; fig. 10 of Burnett 2013) predicts large amounts of SW Mg fractionation, about 10‰/amu. A separate theoretical Mg isotope fractionation prediction of 5–14‰/amu comes from Laming et al. (2017) based on plasma flow through expanding magnetic flux tubes in the lower corona.

Several preliminary values for the solar wind Mg isotopic fractionation are available: Heber et al. (2012b) and Jurewicz et al. (2016) report fractionations consistent the theoretical predictions, but Humayun et al. (2011) find <3‰/amu based on ^25^Mg/^24^Mg.

Given a reliable Mg fractionation, how well can a prediction be made of the O fractionation? It is likely that the theoretical relative fractionations between O and Mg are more reliable than the actual values. Table 2 shows the theoretical relative O/Mg fractionations for the two models, as well as the predicted measured Mg isotopic fractionation if the solar O isotope composition is on the CAI line. Alternatively, empirical mass or square root mass scaling of the 21.5‰/amu required for O can be used to estimate the Mg fractionation. The range of estimated scaling factors between Mg and O fractionations (Table 2) is relatively large; nevertheless, significant constraints are present. For example, if the measured Mg fractionation is zero (Humayun et al. 2011), this would show unambiguously that solar O is not on the CAI line. Beyond this, comparisons of the Laming and Coulomb Drag model predictions with measured solar wind Si and, perhaps, S isotopic compositions should reduce the uncertainties in the relative Mg and O fractionations.

**Table 2:** Predicted O/Mg and Mg isotope fractionations

Model	O/Mg^a^	Predicted Mg^b^
Coulomb Drag	2.8	8
Laming	1.2–1.6	18–13
Empirical^c^		
Linear	1.5	15
Square root	1.2	18

a Relative ‰/amu fractionations.

b For O to be on CAI line (i.e., 21.5‰/amu fractionation).

c Scaling using 25/17 mass ratio.

One of the great advantages of sample return missions is the ability to replicate important measurements. This has not happened with the O isotopic composition. The results of McKeegan et al. (2011) are sufficiently convincing and the amount of work sufficiently large that nobody has stepped forward to do this important task. Our Objective 1 relates to the interpretation of the data, rather than being based on any concern about their accuracy. We have not included replication of the McKeegan et al. (2011) O isotopic composition in our top 12 Science Objectives, but if the Genesis Mg isotopic composition indicated that the solar O isotopic composition was not on the CAI line, then replication would assume greater importance as greater precision would be important.

#### Objective 2: Low (<9 eV) FIP Elements

Uncertainties in photospheric abundances relative to Mg are greater than 10% (1 sigma), and some elements cannot be measured. The Introduction gives a general justification for replacing the CI‐proxy with true solar abundances based on solar wind analyses. As a more specific example, consider the issue of complementarity as it relates to Fe/Mg. Large differences in the composition of “inclusions” (chondrules, CAIs, etc.) and matrix of individual carbonaceous (C) chondrites average out to give a CI bulk composition (e.g., Palme et al. 2015; Ebel et al. 2016). Average inclusion and matrix compositions and proportions among different meteorites and among the different groups of C chondrites differ, but complementarity is maintained. Ebel et al. (2016, p. 344) note: “Why should chondrites be chondritic [i.e., solar]? The answer must supply a first‐order constraint on the origin of chondritic meteorites.” The simplest interpretation of complementarity is that the various C chondrites formed as a closed system in isolated nebular regions. With more valid solar abundances, it is possible to investigate the issue of whether specific C groups, or even individual meteorites, have solar composition. Identifying those groups or meteorites that do not have solar composition potentially provides clues to previously unrecognized or poorly understood nebular processes. The variations of average C group Mg/Si ratios (including CI) are remarkably small, only 6% (fig. 1 of Palme et al. 2015). However, for Fe/Mg, the range is larger: 46% (fig. 2 of Palme et al. 2015), a variation much larger than the errors in the Genesis Fe/Mg, which in turn agrees with the CI ratio.

The goal of Genesis is to improve solar elemental abundance accuracy by a factor of three. But, since we have sampled solar wind and not the photosphere itself, it is necessary to correct for any elemental fractionation between the solar wind and the photosphere. It is important to recognize that mechanisms for *isotopic* fractionation (Objective 1) and mechanisms for *elemental* fractionation are likely different. Fractionations based on first ionization potential (FIP = the energy required to produce a singly charged ion from a neutral atom) are important, but elements with FIP below about 9 eV appear to be unfractionated, based on ±20% 1 sigma data for a few elements from spacecraft solar wind composition instruments.(e.g., Bochsler 2007a; Pilleri et al. 2015; von Steiger and Zurbruchen 2016). Low FIP elements are most of the Periodic Table and are the rock‐forming elements that make up the terrestrial planets. With a few exceptions, present Genesis techniques are sufficiently accurate only for elements lighter than Ni, but among these, there are enough low FIP elements to adequately define low FIP element fractionation.

**Figure 2:**
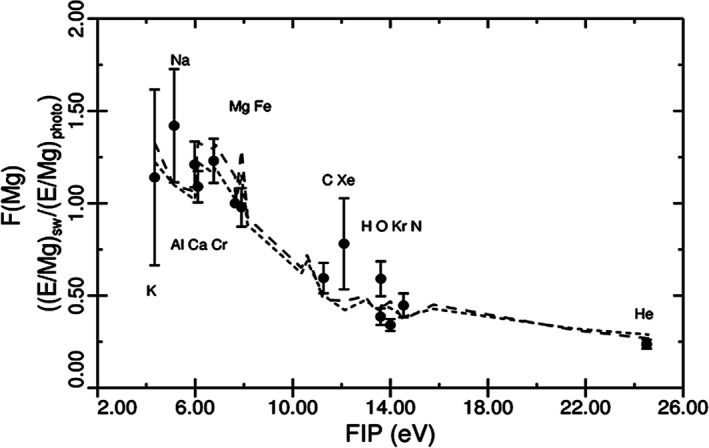
Comparison of fractionation factors from Genesis data with theoretical predictions (dashed lines) based upon models of FIP fractionation by Laming. Figure from Laming et al. (2017). Error bars are 1 sigma. Except for some structure in the Fe‐Mg region that is possibly significant, the two different models of Laming et al. predict essentially the same FIP fractionation trends. References for the individual analyses are given in Burnett et al. (2017).

Genesis elemental abundance measurements are available for 16 elements—including seven low FIP elements by SIMS (ion probe) analyses (Burnett et al. 2017). Fractionation of element E, relative to the photosphere, F(Mg) is defined as:

(1) $$\text{F(Mg)}={\left[\text{E/Mg}\right]}_{\mathrm{SW}}/{\left[E/\text{Mg}\right]}_{p}$$

where abundances are normalized to Mg; SW and p refer to solar wind and photosphere, respectively. Figure 2 shows F(Mg) plotted versus FIP for Genesis‐measured bulk solar wind analyses. Ne and Ar are not plotted, as there are no true photospheric abundances for these elements (but see discussion of Objective 6). The Kr and Xe points are based on interpolation of CI abundances (Wiens et al. 1991). Laboratory implant standards are required for most Genesis analyses; these require calibration, which is feasible but time‐consuming (Burnett et al. 2015). Calibrations have been carried out for all elements plotted in Fig. 2 except for Al and Cr. The fluences for Al and Cr could change by as much as 20%, but all other fluences on Fig. 2 are essentially final.

Figure 2 shows that the theoretical FIP fractionation trends from Laming et al. (2017) provide a reasonable description of the Genesis data. Neither the data nor the theoretical trend matches the expectation from spacecraft instrument data suggesting no fractionation of low FIP elements below around 9 eV. Elements with FIP below 8 eV have F greater than 1 and higher than Fe and Mg. However, at 2 sigma, the data are consistent with lack of fractionation below 9 eV.

The results on Fig. 2 are a significant accomplishment, but much remains to be done. (1) There is strong evidence from spacecraft instrument data that the high speed solar wind is least fractionated (e.g., Bochsler 2007a; Pilleri et al. 2015; von Steiger and Zurbruchen 2016), thus it is important to have the equivalent of Fig. 2 for the Genesis regime samples, especially high speed. Data already exist for He, Ne, and Ar (Heber et al. 2012a), several low FIP elements (Heber et al. 2014b), and H (Koeman‐Shields et al. 2016). (2) We have assumed that fractionation is a smooth function of FIP, but there is evidence both in the Genesis data and in the Laming theory that there is structure in the F(Mg) versus FIP plot. It is important to check for such structure once the final errors are assigned to the data in Fig. 2. (3) Fluences for elements of intermediate FIP (e.g., C, Si, S, P) are important in order to define better the transition to the larger fractionations observed for high FIP elements. (4) The shape of Fig. 2 for low FIP elements, specifically whether or not there is a plateau depends on the elements with the lowest FIP (i.e., Na, K, and Rb). Accurate Na and K fluences are needed.

#### Objective 3: Heavy Element Isotopic Compositions

So‐called UN (unknown nuclear) isotopic variations among inner solar system materials are now known for almost all elements; these may represent nonuniform distributions of refractory presolar grains in an otherwise homogeneous nebula. With the exception of H, O, N, C, and noble gases, these variations are small, usually <1‰. Moreover, with the exception of the same elements, it is unknown whether measurable (greater than about 1‰) systematic isotopic differences exist between the Sun and *all* inner solar system materials. Such differences would indicate a significant violation of the assumption of a homogeneous solar nebula. For example, isotopic variations could result from differences between first materials accreted to the Sun versus the last materials accreted (which may be preferentially present in planetary materials).

Heavy elements are required for this objective because isotopic fractionation during solar wind (SW) formation is minimal at masses heavier than Ar (Heber et al. 2012a). The issue of Earth–Sun differences is intermixed with SW isotopic fractionation for elements lighter than Ar. Any difference between the isotopic composition of a heavy element in the SW compared with that from inner solar system materials would indicate differences in the composition of the nebula from which those materials formed.

Fe is targeted for this measurement objective because of its heavy mass (small SW fractionation), relatively high abundance, and multiple isotopes. Ca, Ti, and Cr are possible alternatives; these elements have larger isotopic variations than Fe among inner solar system materials which may, or may not, indicate larger Sun–inner solar system variations. However, fractionation during SW formation is less of an issue for Fe than for Ca, Ti, or Cr. Fe is highly feasible, allowing for mass interferences, fluences, and surface contamination. This measurement also provides a check on the assumption that the Mg isotopic composition of the Earth and the Sun are very close (Objective 1). Although not heavier than Ar, and potentially subject to small amounts of solar wind fractionation, S is interesting in that it is volatile (Objective 9), and S isotopic variations among meteoritic materials are much smaller than other volatile elements, such as O or N (as reviewed by Chakraborty et al. 2013; their fig. 2).

#### Objective 4: C Isotopic Composition

Lunar rocks are highly depleted in volatile elements, but pulverized surface materials (“regolith”) contain volatiles from a variety of extralunar sources, including the solar wind. Indigenous lunar volatiles, especially H, are a major research area (e.g., McCubbin et al. 2015), but overall, one needs to identify and quantify all inputs. A longstanding lunar puzzle was the large variation of N isotopic composition among lunar regolith samples. Genesis data on the solar wind N isotopic composition (Marty et al. 2011) show that regolith samples typically have a greater amount of extralunar than solar wind N, with the extralunar N having higher ^15^N/^14^N. Füri et al. (2012) concluded that the extralunar N can be accounted for by carbonaceous micrometeorites, but that a major complication is loss of N during impacts followed by partial reimplantation, analogous to the source of excess radiogenic ^40^Ar in lunar regolith samples. If the solar wind ^13^C/^12^C were known, the amounts and isotopic composition of extralunar C in lunar regolith samples could be calculated and compared to the same data for N. Hashizume et al. (2004), based on SIMS analyses of regolith samples, infer a solar wind δ^13^C of −105 ± 20‰. Based on solar CO absorption lines, and using data processing models that give δ^18^O of −50 ± 11‰ (1 sigma error) in agreement with Genesis, Lyons et al. (2018) report solar δ^13^C = −48 ± 7‰. Using a rough estimate of the solar wind C fractionation of 28‰, the inferred solar C isotopic composition from Hashizumi et al. (2004) would be −77‰, possibly acceptably close to Lyons et al. (2018). A Genesis solar wind ^13^C/^12^C measurement could distinguish between −77 and −48‰ and enable improved interpretations of lunar regolith C data.

Beyond lunar science, C is an interesting “intermediate” element in showing smaller isotopic variations than O, N, and noble gases in solar system materials, but larger variations than less volatile elements (Objective 3). Knowing the solar (i.e., average solar nebula) isotopic composition of C would provide a baseline value for the interpretation of C isotopic data on inner solar system materials.

#### Objective 5: Validity of CI Abundances

This objective focuses on providing constraints on the origin of CI chondrites. As discussed in the Introduction, the composition of CI chondrites is consistent with photospheric spectroscopic abundances for many elements (e.g., Palme et al. 2014). CI abundances are a widely accepted proxy for the composition of the photosphere, despite the fact that CI chondrites are petrographically heterogeneous and have been subject to extensive secondary alteration (e.g., Morlok et al. 2006; McSween 1993; Gounelle and Zolensky 2014). Using CI abundances to model how planetary materials formed from the solar nebula is methodologically undesirable and borders on circularity. Genesis data can provide true solar abundances with greater accuracy than photospheric abundances (Objective 2). We focus on Mn, Ga, and Rb because the CI abundances of these elements differ from photospheric abundances by greater than 20% (Palme et al. 2014). Therefore, these elements, although cosmochemically unrelated, may point to unique conditions/processes in the formation history of these important meteorites. Fractionations unique to specific elements are plausible for CI chondrites, and such fractionations have been documented for U (Rocholl and Jochum 1993). Mn is an element with many valence states, including some that would be mobile under oxidizing, aqueous conditions. Rb has lower FIP than Na or K; thus, Rb is important in defining FIP fractionation (Objective 2). Rb also figures importantly in Objective 7. High levels of Ga surface contamination on Genesis samples present a major challenge to the measurement of solar wind Ga.

#### Objective 6: High FIP Element Abundances

Elements with FIP greater than about 9–11 eV are depleted in the bulk solar wind compared to the photosphere by factors of ~2–3, measurable by in situ instruments (e.g., Bochsler 2007a; Pilleri et al. 2015; von Steiger and Zurbruchen 2016). The in situ data show that high speed (coronal hole) CNO are less depleted. von Steiger and Zurbruchen (2016) proposed that coronal hole (high speed) solar wind is not fractionated, thus coronal hole CNO abundances define a heavy element fraction (Z = metallicity) for the Sun that supports the Z from helioseismology and disagrees with the photosphere spectroscopic value (Asplund et al. 2009). This is a decade‐old discrepancy of major importance to solar physics. The von Steiger–Zurbruchen conclusion has been strongly challenged by Serenelli et al. (2016).

As discussed in the Introduction, Genesis collected solar wind in each of three regimes (Neugebauer et al. 2003; Reisenfeld et al. 2013), which reflect different source regions in the Sun (Neugebauer and von Steiger 2001). Separate theoretical regime FIP fractionation factors (equation 1) for each regime, combined with Genesis regime abundances, should independently define the same unfractionated CNO solar abundances. When they do, this will give us great confidence in the derived photospheric abundances for high FIP elements. The Coulomb Drag model (Bochsler 2000) focuses on isotopes and does not deal with FIP fractionation, other than to argue that FIP fractionation is not important for He/H fractionation (see Bochsler et al. 2006). FIP fractionations for high‐ and low‐speed solar wind are predicted from the models of Laming et al. (2017) and these can be refined (see discussion of Objective 2).

H is an important high FIP element, and Genesis data for all regimes are available (Koeman‐Shields et al. 2016). To address the solar metallicity issue, parameters in the theories used obviously cannot be normalized to the spectroscopic photospheric CNO abundances; accurate solar wind regime H fractionation factors thus assume major scientific importance. Good fits of theoretical predictions to the fractionation factors for intermediate FIP elements (e.g., Si, S) will provide confidence in the predictions for high FIP elements.

Excepting He, there are no true photospheric abundances for noble gases, all high FIP elements. The derived FIP fractionation correction needed to obtain a photospheric Kr abundance from Genesis data is important for meeting Objective 7.

Accepting CI abundances, the smoothness of odd A abundance curves for elements heavier than Fe (Burnett et al. 1989; fig. 7 of Palme et al. 2014) allows interpolation of abundances for Kr and Xe (Wiens et al. 1991, 1992; see Objective 7).

**Figure 3:**
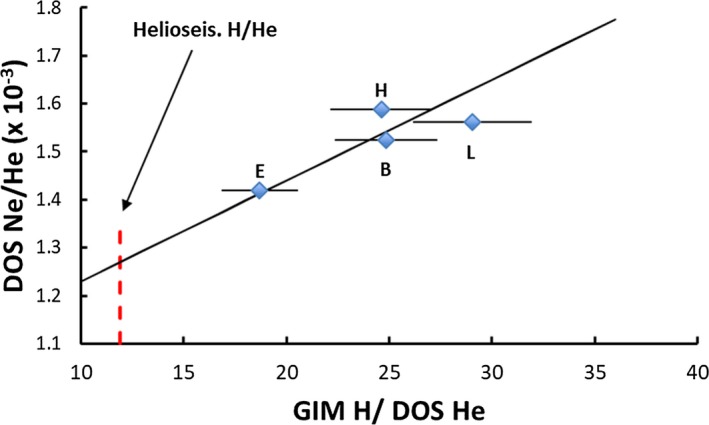
Genesis: (^20^Ne/^4^He) versus (H/^4^He) correlation plot. (E, H, L, B) refer to CME, high speed solar wind, low speed solar wind, and bulk solar wind sample. H fluences from the Genesis Ion Monitor (GIM; Reisenfeld et al. 2013). He, Ne from DOS collectors (Heber et al. 2009a, 2012a; V. Heber, personal communication for E array He and Ne fluences). A visual trend line that fits all data points within the 1 sigma errors is shown. Extrapolation of the trend (triangles) to the helioseismology solar H/He (vertical line; Basu and Antia 2008) gives an estimate of the solar Ne/H (extrapolated Ne/He × solar He/H). [Color figure can be viewed at wileyonlinelibrary.com]

**Figure 4:**
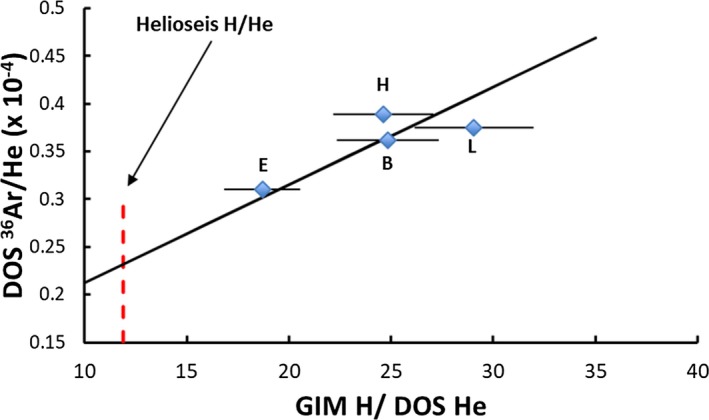
Estimation of solar ^36^Ar/H abundance by same method as for Ne in Fig. 3. E array ^20^Ne/^36^Ar from Heber et al. (2009b). [Color figure can be viewed at wileyonlinelibrary.com]

Abundance curves for elements lighter than Fe are not smooth, so neither the photosphere nor CI chondrites provide abundances for Ne and Ar. Correlation plots involving Genesis regime data for H/He, Ne/He, and Ar/He and the solar H/He measured by helioseismology can be used to estimate photospheric abundances for Ne and Ar (Figs. 3 and 4). H and He fluences are available from the Genesis Ion Monitor (GIM) (Reisenfeld et al. 2013) and He, Ne, Ar data from analysis of diamond‐like‐C collectors (DOS; references in Fig. 3 caption). The GIM and DOS He fluences for the bulk, H, and L arrays agree better than 3%; the E array DOS He fluence is higher than GIM by 12%. The more accurate DOS He fluences are used in Figs. 3 and 4. A linear trend is observed for both figures, but the slope is set entirely by the E array data. Extrapolation of the trends to the solar H/He from helioseismology (11.9 ± 0.2; Basu and Antia 2008) gives estimates of the solar Ne/H and Ar/H (Table 3) that can be compared with literature estimates. Our approach is model independent; it is totally empirical but assumes that extrapolation to the solar Ne/He and Ar/He is valid. Our quoted errors are precision based on the linear fits to the data; we have not estimated systematic errors associated with our approach. Within the quoted errors, the various estimates of Ne/H and Ar/H in Table 3 agree. The Genesis abundances in Table 3 are provisional in that precise DOS H fluences will soon be available (Koeman‐Shields et al. 2016, 2017) allowing the calculation based on Figs. 3 and 4 to be repeated with more accurate data. The empirical solar Ne and Ar abundances yield fractionation factors that can be used to test FIP model predictions.

**Table 3:** Indirect estimates of solar Ne and Ar abundances

Source	Ne/H (10^−5^)	Ar/H (10^−6^)
Genesis^a^	11.5 ± 0.6	2.3 ± 0.5
Asplund et al. (2009)	8.5 ± 2.2	2.5 ± 0.9
Palme et al. (2014)	11.2 ± 2.9	3.2 ± 0.8
Bochsler (2007b)	9.1 ± 3.2	–

a Solar abundances calculated assuming solar ^20^Ne/^22^Ne = 13.6 and ^36^Ar/^38^Ar = 5.47 (Heber et al. 2009a).

Summarizing, Genesis data can provide fractionation factors, not based on CNO, for H, Ne, Ar, Kr, and Xe. The XeKr factors may require accepting CI abundances; The NeAr requires accepting empirical/extrapolated solar abundances (Figs. 3 and 4).

#### Objective 7: A = 77–87 Abundances

**Figure 5:**
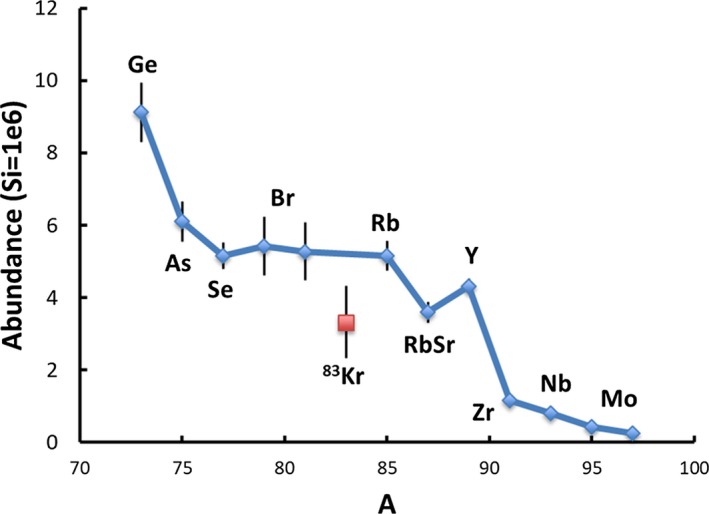
When plotted against the mass number of odd A isotopes, CI elemental abundances form a smooth trend in the Se‐Zr mass region. Interpolation of this trend to mass 83 allows an estimate of the photospheric Kr abundance. The measured Genesis Kr abundance, corrected for FIP fractionation, is distinctly below that estimated from the CI trend. This depletion may indicate preferential accretion of dust relative to gas to the Sun. [Color figure can be viewed at wileyonlinelibrary.com]

CI abundances define a smooth trend when plotted versus the abundance of odd A nuclei (Suess 1947; Burnett et al. 1989). Interpolation of a flat trend in the mass 77–87 region at mass 83 gives an estimate of the photospheric Kr abundance, which cannot be directly measured spectroscopically (Wiens et al. 1991). Kr is a noble gas, depleted in CI. If the Kr abundance estimate, interpolated from mass 77–87 CI abundances, differs from the Genesis‐measured Kr abundance (Meshik et al. 2014), this difference would show that the Sun preferentially accreted either gas or dust, e.g., by preferential incorporation of nebular midplane solids (dust enhancement) or by preferentially accreting gas or ice‐rich planetesimals (gas enhancement). Figure 5 shows that the Genesis solar Kr abundance is significantly lower (3.3 on the Si = 10^6^ scale) than the interpolated Kr abundance (5.2). A correction has been applied for FIP fractionation of a factor of 1.8 based on the measured Genesis fractionation factor for O, which has a FIP close to Kr. The Kr depletion in Fig. 5 would indicate a small amount of preferential accretion of dust to the Sun, but this conclusion is preliminary until corroborated by Genesis data for Se, Br, Rb, Sr, Y, and Zr. Se and Br cannot be measured from photospheric spectra, thus element‐specific fractionations in CI are possible; moreover using Genesis abundances for Se‐Zr eliminates potential complications because of the volatility of some of these elements which may have depleted CI abundances. (Objective 9).

#### Objective 8: Alkali Element Abundances

If solar matter was *partially* ionized at some point during accretion, then it is highly likely that there would be chemical fractionation of ions and neutrals (a cosmochemical case of FIP fractionation). Preferential depletions of easily ionized elements such as Rb, K, and Na would result to the extent that the ionized fraction was lost. Two specific examples:

1. Stellar accretion is observed to occur with the formation of bipolar plasma jets (e.g., Frank et al. 2014) which should produce preferential loss of easily ionized elements, a fractionation that would not show up in SW‐photosphere comparisons, but would be present in comparisons of solar versus CI abundances (Objective 5). Errors on present photospheric Na and K abundances place a limit of around 30% for such ionization fractionations. There is a 64% difference in the CI and photospheric Rb/Si (Palme et al. 2014), but contrary to expectations it is a depletion in CI. Genesis analyses will allow tighter constraints, but amounts of solar wind fractionation of low FIP elements (Objective 2) must be determined first. Although higher accuracy is desirable, measurements of Na and K have been completed (Rieck 2015; Rieck et al. 2016) and were used in Fig. 2. Measurement of Rb, with high mass resolution SIMS instruments, should be feasible, since resolution of ^85^Rb from molecular interferences is possible.
2. It is widely accepted that the formation of the Sun involved loss of magnetic fields; one way to accomplish this is “ambipolar diffusion,” separation of ions and neutrals with ions lost along with the magnetic field (Z. Li et al. 2014 section 3 review the role of magnetic fields in disk formation). Fractionations would be present in all solar system materials and would not show up in CI‐photospheric abundance comparisons. The only test is by comparison with predictions from nucleosynthesis theories. Systematics for average solar system r‐ and s‐process n‐capture are well developed for elements with mass numbers >100, and solar system “r‐only” and “s‐only” abundances are available (e.g., Anders and Grevesse 1989). A plot of r‐only abundances is fairly smooth, and interpolation to Cs, the only alkali metal above mass 100, gives Cs_r_ = 0.745 (atoms/10^6^ Si). S‐only elemental abundances (even‐even nuclei) are not smooth, but σN_s_ is (where σ = astrophysical neutron capture cross section). The analysis of Bisterzo et al. (2014) shows that s‐process contributions to Cs are small (0.052). The predicted total solar system Cs is thus 0.79, over a factor of 2 larger than the CI abundance (0.37), consistent with an ambipolar diffusion depletion of Cs. Our approach is empirical, based only on systematics for average solar system r‐ and s‐processes. We only assume that the average solar system r‐process abundances and σN_s_ are smooth. Although measurement of Cs in Genesis samples does not appear feasible at this time; we hope someone will prove us wrong. The general assessment of CI abundance validity (Objective 5) could help verify this conclusion.

#### Objective 9: Volatile Element Abundances

For elements in the inner solar system, volatility is destiny. In many planetary materials, volatile elements are highly depleted, and amounts of depletion are correlated with estimated condensation temperatures (e.g., Palme et al. 2014). For many volatile elements, photospheric abundances have large errors and, for As, Br, Se, Te, I, Cs, Ta, Re, Hg, and Bi, photospheric abundances are not measurable. Instead, CI‐proxy abundances are used. Although CI abundances are analytically accurate, they are only valid to the extent that they agree with the (at best) poorly known photospheric abundances. Noble gases, H, C, N, and O are not present in solar proportions in CI chondrites, but beyond this, the “volatility cutoff” in CI abundances is not known. The volatile elements listed in Table 1 are selected based on estimated feasibility but are also those most likely to have been lost in the formation of CI chondrites, possibly signifying unique nebula processes. A priori the halogens would be likely to show volatile depletion. Enhancement in photospheric F relative to CIs is possible (Objective 11), so C*l* and Br provide the best tests of volatile depletion in CIs. The photospheric C*l* abundance is uncertain to a factor of two and photospheric Br is not measurable. As discussed with Objective 5, element‐specific fractionation during the formation of CI chondrites is possible. For a related issue, see also the Solar Twins topic in the Discussion section.

#### Objective 10: Li, Be, B Elements, and Isotopes

Relative to CI‐proxy abundances, Li is depleted in the Sun by a large factor (~50×), indicating either (1) destruction (along with D) by thermonuclear reactions in the center of the Sun (this must happen at times before the Sun reached its present configuration where materials from center do not mix to the surface) or (2) destruction at the base of the outer convection zone subsequent to the onset of H‐burning (Oreshina et al. 2015; Boothroyd and Sackmann 2003; Baumann et al. 2010; Thevenin et al. 2017). The observed photospheric Li may reflect (A) planetesimal accretion to the Sun late in solar evolution but early in planetary evolution; here solar wind Li would have the terrestrial planet isotopic composition (^6^Li/^7^Li = 0.08), and would be a significant constraint on models of planet growth (see also Objective 7). Observational evidence exists for the accretion of large planetesimals onto the young star, β‐Pictoris (e.g., Karmann et al. 2003). The required amount of planetesimal accretion to explain the photospheric Li abundance can be estimated: Li concentrations in CI (Palme et al. 2014), CM chondrites (Wasson and Kallemeyn 1988), and models of Earth composition (e.g., Wanke and Dreibus 1988) are similar. We adopt 1.5 ppm. Adopting 3 × 10^31^ g of H in the outer convection zone and the solar Li abundance from Asplund et al. (2009) gives 2 × 10^21^ g of Li in the outer convection zone, equivalent to about 0.2 Earth masses of planetesimals—a number which seems reasonable. The apparent depletion of solar Kr (Fig. 5; Objective 8) indicates a roughly 30% excess of dust, much larger than required to account for the photospheric Li abundance. However, this addition represents nebular processes prior to the establishment of the outer convection zone, with destruction of the Li accompanying this additional dust component.

(B) Alternatively, observed SW Li could be pure ^7^Li, placing a limit to thermonuclear processes which stopped before complete ^7^Li destruction. Finally, (C) the Li could be spallation Li produced by solar flare nuclear reactions subsequent to the establishment of the outer convection zone (compare Objective 11), in which case a SW ^6^Li/^7^Li of about 0.3–0.5 would be expected.

If SW ^6^Li/^7^Li is 0, 0.08, or 0.4, the interpretation is model independent. Intermediate SW ^6^Li/^7^Li between 0 and 0.08 would be subject to model interpretation; however, a SW ^6^Li/^7^Li > 0.08 would indicate spallation input. The combined Li, Be, B data provide more constraints. Photospheric and CI‐proxy abundances agree for Be and B, which is important because it independently constrains the limit of thermonuclear processing. However, large uncertainties in the photospheric abundances of both elements leave room for partial destruction of these elements, producing B isotopic variations. Genesis data for the isotopes of Li and B would permit improved modeling of thermonuclear processes affecting these elements.

#### Objective 11: Radioactive Nuclei and F Abundance

Intense variability in luminosity at all wavelengths (probably accompanied by enhanced emission of stellar winds and flares) is observed to accompany the formation of all stars (e.g., Feigelson 2010; Shu et al. 1997). Our Sun is unlikely to have been an exception. Accordingly, the role of solar proton irradiation in producing some of the extinct short‐lived radioactive nuclei measured in meteorites (e.g., ^10^Be, ^36^Cl, ^41^Ca) is a major issue at present. The extent of such irradiation in the Sun over the last million years can be measured by the abundances of radioactive nuclei (^10^Be, ^26^Al, ^36^Cl and ^53^Mn) in the Genesis lid foils (cf. Burnett et al. 2003; Nishiizumi et al. 2005), which would provide the basis for modeling of the more intense activity in the early Sun. Lunar regolith analysis (Nishiizumi and Caffee 2001) indicates that the amount of ^10^Be in the solar wind is surprisingly high providing support for the early solar wind as a source of extinct ^10^Be measured in meteorites (Bricker and Caffee 2010). The crash badly crumpled and contaminated the large area (8000 cm^2^) Mo‐coated Pt lid foils (Nishiizumi et al. 2005), but techniques are in hand (Nishiizumi, private communication) to flatten and clean the foils. This, coupled with improvements in accelerator mass spectrometry, should enable measurement of all the above radioactive nuclei, possibly excepting ^36^Cl.

The lid foil radioactive nuclei measurements are complemented by measurement of the solar wind F abundance. CI and photospheric F abundances agree, but the photosphere F is uncertain to a factor of 2 (Palme et al. 2014; Asplund et al. 2009). The ^20^Ne/^19^F solar abundance ratio is around 3000, using the CI F abundance. The yield of mass 19 nuclei in spallation of ^20^Ne is high; consequently, a small amount of spallation on ^20^Ne produces a big effect on the F abundance, as illustrated by the ^20^Ne/^19^F in the galactic cosmic rays, which is 5, much lower than 3000 (DuVernois et al. 1996). This large decrease is due to spallation of Ne during interstellar medium transit. Comparison of solar wind and CI F abundances for this purpose requires accepting CI abundances, but at the factor of two level, this is probably not an issue. There are complications as follows. (1) F is a high FIP element (requiring a correction [Objective 6] from solar wind to photosphere), (2) F is a volatile element, and (3) there is no true photospheric Ne abundance. Thus, progress must be made simultaneously on Objectives 6 and 9, but this should be possible. The FIP correction will be about a factor of two; even now, this correction can probably be made to ±20%. Measurements of other volatile elements will further constrain an enhanced SW/CI F ratio. Even at present (Table 2), the photospheric Ne abundance is constrained to within 30%.

#### Objective 12: Heavy/Light Isotope and Element Ratios

The photosphere represents the top of a turbulent outer convection zone in our Sun, comprising approximately the outer 30% of the radius. Beneath the convection zone lies a static mantle in which energy is transmitted purely by radiation. It is possible that heavy elements could sink across the boundary at the bottom of the convection zone, thus changing the composition of the photosphere (“gravitational settling”) (e.g., Michaud et al. 2004; Boothroyd and Sackmann 2003; Piersanti et al. 2007, hereafter PSC). Predicted changes in abundances for elements heavier than C are small, percent levels, unlikely to be detectable in differences between solar and CI abundances. However, the levels of isotopic fractionation predicted by PSC are surprisingly large. PSC predict that gravitational settling has decreased the ^18^O/^16^O ratio by 7‰ over the age of the solar system, but the ratio of Co/Ir has increased by only 6‰ despite the factor of 3 difference in mass.

The predicted O isotope fractionation for gravitational settling is in the same sense as that for Coulomb Drag, so the effects would be additive. The presently inferred difference of 43‰ in ^18^O/^16^O is much larger than the PSC 7‰, but 7‰ is large enough to affect interpretations. Similarly, the PSC prediction of 4‰ is significant compared to the 20‰ Coulomb drag prediction for fractionation of ^26^Mg/^24^Mg (Objective 1). SW isotopic fractionation dies out rapidly with increasing mass; thus, the best tests are in the Ca‐Fe region. PSC predict 1‰/amu fractionation for Ca isotopes. Because of the low abundance of ^48^Ca, measurement of ^48^Ca/^40^Ca fractionation in Genesis samples is probably not feasible; but, although difficult, the predicted 4‰ fractionation in ^44^Ca/^40^Ca or the predicted 1.6‰ fractionation in ^56^Fe/^54^Fe might be measurable. It is more likely that further evaluation of the PSC predictions must come from theoretical studies.

Theories can be wrong, and independent observational tests of gravitational settling are important. Use of Genesis data to test for gravitational settling requires adopting CI‐proxy abundances as the initial photospheric abundances (independently tested by Objective 5). To minimize errors from the adoption of CI abundances, we propose comparing heavy and light siderophile elements, which would tend to be unfractionated in CI chondrites *as a group*. (Siderophile elements are chemically similar, concentrating into meteoritic metal phases.) Ni and Co are the best light siderophile elements. A measurement of the Ni fluence has been made (Choi et al. 2016). Ir is currently the most feasible heavy siderophile element to measure, although data on others (such as Re or Os) are highly desirable.

### Appropriate Collector Materials for Measurement Objectives

As indicated in the Introduction, Genesis flew a variety of collector materials (Burnett et al. 2003; Burnett 2013; Jurewicz et al. 2003). There were two main types: Concentrator targets and collector array samples. Other, single material collector instruments (Jurewicz et al. 2003), as well as exposed spacecraft parts, contain bulk solar wind and are available for analysis through Genesis Curation. For all measurements, it must be verified that (1) any cleaning removed contamination but did not remove solar wind and (2) that the impurity levels for elements of interest in the collector material itself are sufficiently low. An overview of flight collector materials is given in Jurewicz et al. (2003); their post‐return availability is reviewed in Gonzalez et al. (2015) or the JSC Genesis Web Page https://curator.jsc.nasa.gov/genesis/index.cfm.

#### Measurements Using Concentrator Targets

Table 4 gives examples of analyses suited to using Concentrator samples. Given clean surfaces, all of these measurements are feasible using small‐area analytical techniques.

**Table 4:** Isotopic and Elemental Measurements Feasible with Concentrator Targets

Element	Fluence (cm^−2^)^a^	
Li^b^	4 × 10^7^	SiC target purity verified. Development needed.
Be	3 × 10^7^	Target purity yet to be established. Development needed.
B	8 × 10^8^	Target purity yet to be established. Development needed. Potential serious contamination issues.
C^c^	1 × 10^14^	Isotopes probably feasible on bare Si of DLC quadrant (SIMS) (Rodriguez et al. 2009; Allton et al.^2013^).
N	4 × 10^13^	Analysis complete (Marty et al. 2011).
O	2 × 10^14^	Analysis complete (Mckeegan et al. 2011).
F	1 × 10^10^	Target purity yet to be established.
Ne	4 × 10^13^	Analyses done and used for modeling Concentrator fractionation (Heber et al. 2007, 2011).
Mg	4 × 10^13^	Isotopes feasible on array materials. Then Mg isotopes can be used to obtain more accurate Concentrator fractionation.
Si^d^	4 × 10^13^	Measurements likely possible (DLC and ^13^C diamond quadrants)Target purity is yet to be established. Very likely feasible (eg., SIMS) as ^13^C diamond was grown from gas phase and likely has a low background.
P	2 × 10^11^	Target purity yet to be established, but likely not an issue.
S	1 × 10^13^	Target purity yet to be established, but probably not an issue. Errors minimized by analyzing “sweet spot” along radius where isotopic fractionation is minimum.
Cl	6 × 10^10^	SiC and ^13^C diamond. Target purity yet to be established.

a Based on average concentration factor of 20.

b LiBeB require advanced instrumentation.

c C and F require no new instrumentation.

d Si, P, S, Cl may require additional analysis of Concentrator isotope fractionation (see Mg this table).

Elements from Li to Mg are quantitatively focused by the Concentrator mirror electrode (Wiens et al. 2013), but collection efficiency drops off for elements heavier than Si. Elements heavier than Ca cannot be measured in Concentrator targets. The fluence and isotopic composition of Ar in Concentrator targets gives 3.5%/amu instrumental mass fractionation (CIMF) (Heber et al. 2007, 2011). Modeling optimized to fit the Ar data by Wiens et al. (2013) enables the feasibility estimates in Table 4. The measured CIMF for Ne (Heber et al. 2007, 2011) provides the basis for correcting measured Concentrator O and Mg isotopic compositions. The solar wind Mg isotopic composition, independently measured on array materials (Objective 1), could be used to refine models of the Concentrator collection efficiency and CIMF.

#### Measurements Using Collector Arrays

The collector arrays sampled solar wind without fractionation during collection except for backscattering, for which accurate, small corrections are possible using SRIM. Different arrays were deployed depending on solar wind regime, as selected by the solar wind monitors (Barraclough et al. 2003). The bulk solar wind fluence is 20 times lower on average than for light elements in the Concentrator targets. Regime fluences are lower, but known (Reisenfeld et al. 2013). Serious fragmentation, contamination, and material‐losses were caused by the crash; however, the regime of fragment is known from its thickness.

Silicon is the easiest material for Genesis PIs to use because of its long history in the semiconductor industry, which includes cleaning and analytical techniques. For Genesis, advances have been made, especially SIMS backside depth profiling (Heber et al. 2014a, 2014a). Unfortunately, more than three‐fourths of the silicon collector material was “lost” (fragments <10 mm^2^) in the crash. Diamond‐like‐carbon on Silicon (DOS) collectors have been backside‐thinned for SIMS analyses and then backside implanted to give internal standards (Rieck 2015). However, for Mg and Na, SIMS sensitivity changes with position (Rieck 2015; Jurewicz et al. 2017a, 2017b). Other than SIMS analyses, DOS is excellent because collectors, although broken, are relatively unscratched and are relatively easy to clean because of resistance to chemical attack. DOS does, however, have variable nonuniform impurity levels. Only a few mm‐sized Ge collector fragments were recovered. Sapphire (Al_2_O_3_) collectors are hard so that their surfaces are relatively undamaged; they are chemically resistant, so can be aggressively cleaned. Some large samples of coated sapphire collector materials (Au, Al, Si) were recovered, but these have suffered extensive surface damage, and cleaning is difficult. The Si coating on the SoS collectors does not completely stop the solar wind and has been heavily oxidized, so cleaning is difficult (Humayun et al. 2011).

Either small‐area (<500 micron) analyses need to be made with advanced analytical techniques or techniques for large area (>50 mm^2^) analyses must be developed that, if necessary, “stitch” together analyses of multiple small collector fragments. New techniques and instrumentation, e.g., resonance ionization mass spectrometry (RIMS), need to be developed for Genesis in order to work with limited amounts of material. Even where not needed for a specific measurement, advanced analytical techniques would more effectively utilize samples. New, commercial TIMS mass spectrometers potentially can make an Fe isotopic measurement on 2 cm^2^ of Genesis array silicon versus the 64 cm^2^ of array silicon needed for analysis with current instruments (Sharma 2016). Another profitable approach is to focus on materials other than Si which are more readily available.

## Discussion

### Genesis and the Homogeneous Nebula Assumption

Much of the above discussion is based on the (commonly made) assumption that planetary materials formed from a homogenous solar nebula, the composition of which is preserved for us in the solar photosphere (with the exception of D, ^3^He, and Li). Several Genesis Objectives test the assumption of a homogeneous nebula.

Compositions of outer solar system materials are poorly known. The assumption of a homogeneous nebula may be limited to inner solar system materials. The Stardust mission returned samples from Comet Wild 2; however, it appears that most, if not all, of the larger Stardust grains, and probably the silicate portion of all short period (Jupiter family) comets, are high temperature inner solar system materials (e.g., Brownlee 2014).

Leaving aside the close similarity of CI and photospheric spectroscopic composition, nebular homogeneity is supported by the ubiquity of the “chondritic” composition and by the overall uniformity of isotopic compositions at the ‰ level for most elements.

Potential violations of the nebular homogeneity assumption are (1) *extant* presolar grains and (2) sub‰ isotopic variations, e.g., in Cr and Ti (Warren 2011) or Mo (e.g., Budde et al. 2018) which may also derive from an inhomogeneous distribution of *assimilated* presolar grains. Both (1) and (2), although very important, can be regarded as minor exceptions to the nebular homogeneity assumption. There is a major literature on whether short‐lived radioactive nuclei (e.g., ^26^Al) were homogeneous in the solar nebula. If inhomogeneous, such isotopes may have been injected relatively late and may be inhomogeneous because they failed to mix with an otherwise homogeneous solar nebula. This is a separate discussion from what we are considering here.

The large isotopic variations among terrestrial planet materials for O, N, H, and noble gases are apparent failures of the nebular homogeneity assumption. But, to the extent that O, and possibly N, isotopic variations are due to self‐shielding (Lee et al. 2008; Nuth et al. 2014; Lyons 2012; X. Li et al. 2014), these variations represent a solar system process acting on an isotopically homogenous nebular gas, i.e., self‐shielding models assume an initial homogeneous solar nebula with solar isotopic composition.

Large variations in H and N isotopes in outer solar system materials (e.g., Füri and Marty 2015 and references therein) suggest that the nebular homogeneity does not apply to the outer solar system, but Füri and Marty (2015) argue that the variations are due to solar nebula processes: ion molecule reactions and self‐shielding. Larger isotope enrichments in D (factor of 200 relative to solar) and ^15^N (up to a factor of 5 relative to solar) are associated with “hot spots” in meteoritic and interplanetary dust carbonaceous material (e.g., Alexander et al. 2017). Ion molecule reactions are commonly invoked to explain such large isotopic fractionations; to the extent that these could occur in colder regions of the solar nebula, the large isotopic variations in H and N are compatible with the nebular homogeneity assumption. The hot spot carbonaceous host phases may be cometary. The carriers of the large N and H isotopic anomalies were inhomogeneously distributed throughout the solar nebula, but this is not necessarily a failure of nebular homogeneity. Rather, it could represent localization of the solar system processes causing the isotopic variations, probably in the outer solar system, followed by radial mixing inward into at least the asteroid belt. The C isotopes in these materials are not equally anomalous, but variations approaching 100‰ are present and not easily explained; these can be better interpreted in the context of a Genesis solar C isotopic composition (Objective 4).

The relationship between inner solar system noble gases and SW noble gases is reviewed in Burnett (2013). The relevant comparison is between solar wind and the chondritic “trapped” noble gas component, “Q,” which is a surface correlated (adsorbed?) component on the surface of acid‐insoluble organic carbon (e.g., Busemann et al. 2000). Does Q represents mass fractionated solar nebula noble gases or is it a presolar component?

Solar wind He has large ^3^He enrichments from solar D burning. Adopting the Galileo probe Jovian ^3^He/^4^He (Taylor et al. 2004) as the solar nebula ratio, the Q‐solar He fractionation is 139 ± 5‰ with a depletion of ^3^He in Q. The Genesis bulk ^20^Ne/^22^Ne is 13.8 (e.g., Heber et al. 2009a). The Bochsler (2007b) Coulomb Drag model predicts that the Ne/O fractionation ratio should be 0.57; combining this with the 21.5‰/amu fractionation for O from McKeegan et al. (2011) gives an estimated Ne fractionation of 12‰/amu corresponding to a solar ^20^Ne/^22^Ne of 13.6 with a maximum error of around ±0.2. Although there are well‐documented intersample variations in Q Ne (Busemann et al. 2000), these all lie in the range 10.4 ± 0.4, well below the solar ^20^Ne/^22^Ne. The Q‐solar Ne fractionation is thus 118 ± 4‰/amu, surprisingly not much less than for He. The Q‐solar Ar fractionation is 14 ± 2‰/amu, much less than for Ne.

For Kr and Xe, solar wind, solar isotopic fractionation should be negligible. If Q Kr and Xe are mass fractionated solar nebula gas, then the ‰ differences between Genesis and Q should be a linear function of mass, which is not observed (Burnett 2013; his figs. 15a and 16a based on Meshik et al. 2014). The deviations from a linear (Q‐solar) trend are interpreted as contributions from Xe HL and possibly other presolar grain components to Q (e.g., Gilmour 2010; Meshik et al. 2014). Linear portions of the (Q‐solar) trend in the mid mass range for Kr and Xe can be used to define the fractionation factors, as summarized in Table 5.

**Table 5:** (Q‐solar) fractionation factors (light isotope depleted)

	‰/amu
He	139 ± 5
Ne	118 ± 4
Ar	14 ± 2
Kr	9^a^
Xe	10^a^

a Burnett (2013, table 2).

The fractionation factors in Table 5 test the hypothesis that Q noble gases formed by mass fractionation from a homogeneous solar nebula of solar composition. The trends in Table 5 are not especially satisfactory; the drop between He and Ne appears too small, that between Ne and Ar appears too large, and a higher degree of mass fractionation for Xe than Kr may be unphysical. These anomalies may indicate that Q is a presolar component, having no genetic relation to solar noble gases, a failure of the nebular homogeneity assumption. But it may be that a variety of fractionation processes are at work and that Q, although formed by solar system processes, is—to an even greater degree than already assumed—not a single component.

A dedicated advocate could defend nebular homogeneity in spite of the large isotopic variations in many volatile elements. It is important to define further the limits and failures of the nebular homogeneity assumption. Several of the Objectives in Table 1 provide tests of nebular homogeneity, specifically Objectives 3, 7, 8, 10, 12, and possibly 1 if the solar O isotopic composition does not lie on the CAI line (Fig. 1). If there was major mass loss of partially ionized matter from the Sun in the form of bipolar jets, it seems inevitable that there would be chemical consequences; if this happened, the solar nebula may have been homogeneous, but its average composition is not solar. It is quite possible that these are the most important Objectives.

### Genesis and Solar Twins

Considerable interest exists about the possibility of using small (% level) abundance *differences* between the Sun and stars of very similar size, luminosity, and overall composition (“solar twins”) to detect the presence of planet formation. The (plausible) assumption is that *relative* abundances for a given element can be measured more precisely than the abundances themselves (e.g., Melendez et al. 2017). Chambers (2010) proposed that ≈5% abundance differences between the Sun and solar twins represented a depletion of refractory relative to volatile elements in the Sun and that this depletion reflected the formation of the terrestrial planets at a time subsequent to the establishment of the solar outer convection zone. The Sun and solar twins formed at different times. Thus, the interpretation of small compositional differences is complicated by effects of galactic chemical evolution, and the compositional differences can alternatively be due to age differences (e.g., Tucci Maia et al. 2016). The Chambers effect could potentially show up as volatility‐correlated differences between CI and Genesis abundances (Objective 9), but differences at the 5% or smaller level are difficult to detect. The apparent 34% depletion of Kr (Fig. 5) is in the opposite direction and much larger than the Chambers effects, perhaps reflecting processes earlier in solar accretion.

### Important Measurements Not Currently Feasible

Important measurements, some planned prelaunch, may be unachievable because of the crash. However, as new techniques will surely become available with time, they are worth mentioning (1) complete REE patterns, (2) Th and U fluences, (3) Pb isotopes, and (4) Cs fluence.

1. Deviations of REE patterns from the “chondritic” pattern provide important interpretations of geochemical and cosmochemical processes. The chondritic pattern is well defined for carbonaceous and ordinary chondrites, and has value as a reference to the extent that the homogeneous nebula assumption is valid. High mass resolution SIMS instruments (Cameca 1280‐1290 series) may be able to measure Ce and La (Ce = 3 × 10^6^ atoms/cm^2^). Radiochemical neutron activation coupled with low‐level counting may be able to measure selected REEs. One way to measure a complete pattern is through resonance ionization mass spectrometry (RIMS). However, RIMS requires at least two lasers per element; doing all 14 REE would be time‐consuming.
2. Many ratios of refractory lithophile elements serve as planetary constants in that they tend to be preserved in igneous differentiation because of their incompatibility. Th/U, K/U, and K/Th are important examples. U is inhomogeneously distributed in CI chondrites (Rocholl and Jochum 1993), but equilibrated ordinary chondrites provide a good estimate of the solar system Th/U (Goreva and Burnett 2001). If the true solar Th/U value differed from ordinary chondrites, this would point to unique solar nebula processes. There are only upper limits on photospheric Th and U abundances. K/U is remarkably constant in lunar samples and distinctly lower than the Earth, which is in turn lower than the chondritic ratio (e.g., Taylor 1993). K/Th can be measured by remote sensing gamma counting on planetary surfaces, but a true solar ratio is needed for comparison. Measurement of the Genesis K fluence is clearly feasible (Rieck 2015; Rieck et al. 2016), but the fluences of U and Th appear too low in Genesis samples (1.7 × 10^4^ and 6.3 × 10^4^ atoms/cm^2^).
3. The Pb isotopic composition of Canyon Diablo troilite has served as the accepted initial Pb isotopic composition of the solar system for half a century. A true solar value is highly desirable. Based on CI Pb and U concentrations and Pb isotopic composition (Baker et al. 2010) and an iron meteorite troilite Pb isotopic composition (Toluca; Nielsen et al. 2006), the solar ^206^Pb/^204^Pb ratio contains about 1.5% radiogenic Pb, sufficiently small to permit determination of the solar initial Pb isotopic composition. The measurement is very difficult. Total Pb is estimated at ~6.0 × 10^6^ atoms/cm^2^.
4. As discussed for Objective 8, combining the CI abundance of Cs and estimates of the solar system r‐only and s‐only abundance patterns would support the possibility of Cs depletion due to preferential loss by ambipolar diffusion during magnetic field loss in the formation of the solar system. A measurement of the Genesis Cs fluence (~6.9 × 10^5^ atoms/cm^2^) would provide a better basis to constrain this important evolutionary event.

### Radiation Damage

Despite H suppression (Wiens et al. 2013), H fluences near the center of Concentrator targets are high (≈10^17^/cm^2^) and bubble formation in exposed Si, the first few mm from the center of the diamond‐like C (DLC) target, is observed. Collector array materials have lower (≈10^16^/cm^2^) H fluences. Radiation‐induced segregation of Mg in silicon has been inferred. In DLC, localized structural radiation‐induced changes may be present (Jurewicz et al. 2017a, 2017b). These changes do not cause loss of solar wind, but in Si they may change the distribution of elements in the radiation‐damaged zone, and if the segregation is close to the surface, solar wind could be misinterpreted as surface contamination. The radiation‐damaged matrix could also locally change sensitivity factors in SIMS analyses. These issues differ from sensitivity variations caused by minor components of the matrix because they are not uniform with depth. Accordingly, a simple monoenergetic implant to check for the effect of H on SIMS or RIMS sensitivity factors may not identify radiation damage problems. Accurate simulation of the amount and depth distribution of the solar wind H radiation damage using monoenergetic ion implants is difficult.

### Need for Redundancy of Measurement

Ideally, high priority measurements need to be performed using multiple techniques and/or in different laboratories, preferably using different types of solar wind collectors, but the amount of effort to meet this ideal is large. Not every collector material was tested for the retention of every component of the solar wind. The solar wind Na measurements by Rieck (2015) and Rieck et al. (2016) illustrate the importance of redundancy. SIMS measurements were performed using the same internal standard technique based on implants of actual flight samples, but using two collector materials. The results separately for both Si and DLC were reproducible in two laboratories; however, the measured solar wind Na fluence from silicon collectors consistently differed from that in diamond‐like carbon collectors by a factor of ~2. The deviation was in the wrong direction for Na diffusion loss from Si (unless Na had diffused into the collectors from the surface). This result is consistent with inhomogeneity of DLC (Jurewicz et al. 2017a, 2017b), but was not something that would have been expected a priori.

### Practical Issues Relevant to Genesis Sample Analysis

#### Accuracy

Compared to most missions, Genesis has the challenge of making precise quantitative analyses instead of qualitative observations. But Genesis also has the major advantage that the measurements are being made in terrestrial laboratories rather than during flight. Quantitatively, isotope analyses at the ‰ or better level for major isotopes is required. The elemental analysis goal is to improve on Mg‐normalized abundances by a factor of 3 in accuracy relative to spectroscopic abundances.

#### Completeness

Table 1 is our assessment of the *minimum* amount of future science return. The prioritization reflects our personal experiences. All Objectives represent important science worthy of funding. Development of new approaches and/or instrumentation will inevitably happen, providing the means to address important problems that are not yet feasible.

#### Large Area Analyses and Surface Contamination

Analyses are more difficult because of the crash, due to contamination and sample loss. Techniques such as laser ablation extraction (for noble gases) and SIMS successfully analyzed sub‐mm areas on Genesis samples. Extraction of solar wind from larger areas could give many more atoms per analysis, allowing analysis of low‐abundance elements. Precrash, experiments were planned with sample areas as large as 50 cm^2^; now the largest areas available are around 1 cm^2^ (Allton et al. 2005; Burkett et al. 2011; Gonzalez et al. 2015). Still, many analyses are possible with 0.5–1 cm^2^, and our feasibility assessments assume no larger areas. The key to analyzing cm^2^‐sized areas is to have clean surfaces. Characterization and cleanliness verification techniques have been developed which do not disturb the solar wind profiles (e.g., Goreva et al. 2014, 2015, 2016; Kuhlman et al. 2016; Allton et al. 2007, 2016; Schmeling et al. 2012; Janakiraman et al. 2018; Welten et al. 2018); however, the surface contaminants are complex and difficult to remove. Hot aqua regia applied to Si collectors has been most successful thus far (Waeselmann et al. 2015). Although much more work is needed, the key point is that the contamination is on the surface, but the solar wind is safely beneath the surface.

An in‐flight polydimethyl‐siloxane contamination film (affectionately referred to as “brown stain”) is present in variable amounts on collector array samples. This was recognized as a visible discoloration on Sun‐exposed Al surfaces from the sample canister (Burnett et al. 2005). FIB‐TEM measurements on a gold foil sample showed a thickness of about 5 nm (Calaway et al. 2006). On Si collector materials, ellipsometry estimates of brown stain thickness are 2–6 nm (Calaway et al. 2006) but these are uncertain. Photoelectron spectroscopy (XPS) analyses on Si (based on C) or diamond‐like carbon (based on Si) give brown stain thicknesses <2 nm with many samples having no detectable brown stain. Although present, the brown stain appears sufficiently thin to have negligible effect on Genesis sample analysis, unlike the crash‐derived contamination.

#### Innovation/Development/Feasibility

These are intimately related. Improvement in analytical techniques will continue, e.g., SIMS backside depth profiling (Heber et al. 2014a, 2014b) greatly minimizes the effect of contaminated surfaces and has permitted good analyses of Ca and Al, elements that have high levels of surface contamination (Heber et al. 2014b). “Development” need not necessarily mean multimillion dollar laboratory instruments, but instead, relatively low‐tech innovations, e.g., individual pixel analysis of SIMS rastered beams during backside depth profiling to eliminate the effects of small particulate contamination (Westphal et al. 2014). Nevertheless, with rare exceptions (Br, Kr, Xe [Meshik et al. 2014], and Ir), the analysis of elements heavier than Fe will likely involve advanced instrumentation having lower detection limits, e.g., RIMS.

#### The Special Role of Concentrator Samples (Table 4)

The target of the Concentrator was ~25 cm^2^, divided into four (~6 cm^2^) quadrants: two of SiC, one of ^13^C chemical vapor deposition (CVD) diamond, and one of diamond‐like carbon (DLC). The SiC and ^13^C diamond are intact, and only small areas of one SiC concentrator target were used in doing O and N isotopic analyses. The DLC target is broken, but mostly available (Rodriguez et al. 2009). The targets have on average 20 times more fluence than the collector arrays, and have less surface contamination. They are chemically and physically resistant and therefore relatively easy to clean the concentrator quantitatively captured ions for elements lighter than Mg and partially captured elements up to about Ca (Wiens et al. 2013).

#### Calibration: An Opportunity for Immediate Participation

All applicable analytical techniques require standards. Procuring and/or fabricating standards for SW calibration has turned out to be a major problem for secondary ion mass spectrometry (SIMS) analysis, but significant progress has been made (Burnett et al. 2015; Heber et al. 2014a). The semiconductor industry produces ultraclean materials. Although good for Genesis collectors, it (ironically) is difficult to find appropriate materials with known and uniform impurity concentrations (e.g., Mg in Si) for standards. Calibrating an implant standard using Rutherford backscattering (RBS) works well for elements heavier than Na and for fluences ~5 × 10^15^ atoms/cm^2^ or greater. These fluences are very high relative to most SW elements; an RBS‐calibrated standard requires at least one intermediate standard before SW can be measured accurately. This has been successfully done in measuring the Fe fluence, but requires a major amount of effort. In any potential standard, the element of interest must be shown to be uniformly distributed. Making calibrated, low‐concentration analytical standards is one area where experimental petrologists and others could contribute directly to the Genesis effort and perhaps to the semiconductor industry as well.

#### Finding Information on Genesis‐Specific Work

Recent Genesis work, especially on cleaning, is primarily found in LPSC abstracts (e.g., Goreva et al. 2014; Kuhlman et al. 2013, 2016; Janakiraman et al. 2018; Welten et al. 2018) or as files on individual samples held by the Genesis curator at JSC or by the authors of this paper. These are available upon request. Much current work addresses issues such as removing contamination without damaging the solar wind layer. To share information, an annual meeting (Genesis Samples Users Meeting), open to everyone, is held in Houston the Sunday before LPSC. We plan to continue this meeting as a resource for information on Genesis status.

## Conclusions

Genesis analyses were always going to be hard. Many standard techniques have insufficient sensitivity or have sample processing blanks that are too high. The crash increased this difficulty. Progress on the goal of providing accurate (elements) and precise (isotopes) solar compositions based upon the Sun, not a meteorite, has been slow, but steady. Much work up to the present has been the unglamorous tasks of understanding how to work with fragmented and contaminated samples. To mitigate the effects of the crash, Genesis scientists have developed new sample handling and analytical techniques, reduced blanks, and increased instrument sensitivity to accommodate smaller samples. The stage has been set for future high‐impact science, as outlined in Table 1 and in the Discussion. The prioritization involved in Table 1 should not be given great weight; any of the 12 objectives that are ready to go should be carried out. For the right PI, there is still great Genesis science waiting to be proposed. The general science objectives have not changed; however, we have become more pragmatic in allowing for smaller samples than anticipated and for contamination. The amount of time and effort depends upon the element being measured as well as the collector, but in some cases, all that is required is to develop better analytical standards. In other cases, e.g., Fe and S isotopic analyses, samples cleaned completely of surface contamination over cm^2^ areas are required. Finally, in cases like Se and Sr abundances (Objective 8) and heavy elements in general, new instrumentation is required.

In spite of analytical challenges, we conclude that obtaining improved solar elemental and isotopic abundances from solar wind (SW) data remains feasible, as well as scientifically important. We have safely returned solar matter to Earth. Although we are hurt by damage in the crash, as a sample return mission, we potentially have the capability to overcome these difficulties. Extracting information on the nebula from solar wind is obviously iterative, but we have the power to iterate. A key issue is whether SW–Sun fractionations are smooth functions of FIP so that we can interpolate corrections. At a minimum, a good assumption is that elements with nearly the same FIP, e.g., Fe and Mg, are not fractionated. We rely on error estimates of the spectroscopic abundances, but these have been carefully evaluated. Theoretical fractionation models are of immense importance, as well as the recent refinements of in situ instrument solar wind composition data (e.g., Pilleri et al. 2015).

The relationship of Genesis to CI abundances has nuances. Ideally one would want to replace CI abundances by an accurate set of true solar abundances, but this is probably not realistic for the whole Periodic Table. Historically, as the accuracy of spectroscopic photospheric abundances has improved, the agreement with CI abundances has improved. We have no insight into whether photospheric abundances can be significantly improved; the conflict with helioseismology over solar metallicity (see Objective 6 discussion) appears to be a stalemate at present. Beyond this, it is not obvious that there is sufficient motivation for a solar physicist to work to reduce the errors on random elements, although Genesis results could inspire attention to specific elements (see discussion on Objective 5). As noted in the Introduction, the *validity* of CI abundances is limited by the errors in solar abundances; the goal of Genesis is to use solar wind abundances with smaller errors in place of the spectroscopic photospheric abundances, thus testing the validity of the CI abundances. In practice, the role of Genesis likely will be not to repeal and replace CI abundances, but to either better establish their validity or to reveal deviant abundances, thus providing more insight into the origin of these important materials.

## Summary

Initial Genesis accomplishments were reviewed by Burnett (2013) and are updated here. Additional important, but feasible, science is presented in the form of 12 Science and Measurement Objectives (Table 1) with Objective by Objective rationales given in subsequent sections. Although prioritized, all Objectives are important, independent, and waiting impatiently for the right PI. Sequential accomplishment of Objectives is not required.

The individual Objectives are independent studies, but there are general themes. In situ spacecraft data showed that *elements* in the solar wind are fractionated from the photosphere; Genesis (Heber et al. 2012a) proved that *isotopes* are also fractionated during solar wind formation. Quantitative measurements of these fractionations are the focus of Objective 1 (for isotopes) and Objectives 2 and 6 (for elements). Considerable progress, both analytical and theoretical, has been made on elemental fractionation (Fig. 2). Searching for systematic isotopic differences between the Sun and inner solar system materials is the theme of Objectives 3 and 4. Tests of the limits to which CI elemental abundances are a valid proxy for solar abundances is the focus of Objectives 5 and 9. Several important Objectives (3, 7, 8, 10, and 12) test the assumption that planetary (at least inner solar system) materials formed from a homogeneous solar nebula, the composition of which is stored in the solar photosphere. Objective 11 is a unique study focused on the effects of intense early solar flare and solar wind exposure by constraining the million year and billion year average solar particle fluences, an issue of major relevance to early solar system time scales based on extinct radioactive nuclei. Objective 12 tests the extent of gravitational settling in the Sun.

## Editorial Handling

Dr. Marc Caffee
